# Examining Road Traffic Mortality Status in China: A Simulation Study

**DOI:** 10.1371/journal.pone.0153251

**Published:** 2016-04-12

**Authors:** Helai Huang, Qingyi Yin, David C. Schwebel, Li Li, Guoqing Hu

**Affiliations:** 1 Urban Transport Research Center, School of Traffic and Transportation Engineering, Central South University, Changsha, 410075, China; 2 Department of Psychology, University of Alabama at Birmingham, Birmingham, 35294, United States of America; 3 Department of Epidemiology, School of Public Health, West Virginia University, Morgantown, West Virginia, United States America; 4 Department of Epidemiology and Health Statistics, Xiangya School of Public Health, Central South University, Changsha, 410078, China; Beihang University, CHINA

## Abstract

**Background:**

Data from the Chinese police service suggest substantial reductions in road traffic injuries since 2002, but critics have questioned the accuracy of those data, especially considering conflicting data reported by the health department.

**Methods:**

To address the gap between police and health department data and to determine which may be more accurate, we conducted a simulation study based on the modified Smeed equation, which delineates a non-linear relation between road traffic mortality and the level of motorization in a country or region. Our goal was to simulate trends in road traffic mortality in China and compare performances in road traffic safety management between China and 13 other countries.

**Results:**

Chinese police data indicate a peak in road traffic mortalities in 2002 and a significant and a gradual decrease in population-based road traffic mortality since 2002. Health department data show the road traffic mortality peaked in 2012. In addition, police data suggest China’s road traffic mortality peaked at a much lower motorization level (0.061 motor vehicles per person) in 2002, followed by a reduction in mortality to a level comparable to that of developed countries. Simulation results based on health department data suggest high road traffic mortality, with a mortality peak in 2012 at a moderate motorization level (0.174 motor vehicles per person). Comparisons to the other 13 countries suggest the health data from China may be more valid than the police data.

**Conclusion:**

Our simulation data indicate China is still at a stage of high road traffic mortality, as suggested by health data, rather than a stage of low road traffic mortality, as suggested by police data. More efforts are needed to integrate safety into road design, improve road traffic management, improve data quality, and alter unsafe behaviors of pedestrians, drivers and passengers in China.

## Introduction

Road traffic crashes are the 8^th^ leading cause of global deaths[1], causing major international public health bodies such as the United Nations to call for global action to reduce road traffic crashes [2]. As the country with the largest population in the world, China is particularly vulnerable to the impact of road traffic injury[3, 4, 5, 6], and concerted efforts to improve road traffic safety in China have the potential to greatly reduce global road traffic injury rates. Accurate assessment of road safety data is imperative for the implementation and evaluation of preventive strategies.

There are two primary sources of road traffic mortalities in China, police data and health department data. Currently, the two data sources present conflicting mortality rates and trends [7, 8]. In one study, police data were reported to underestimate road traffic mortality by about 50% compared to health department data [7]. In a second study comparing road traffic deaths in 2009 police data to WHO estimates, police data indicated about 45% fewer deaths than WHO estimates [8]. Considering the extensive use of police data for the formulation and tailoring of road traffic safety policies in China [9, 10, 11], it is urgent to assess the accuracy of the police data, along with alternative data sources such as the health data. In particular, two critical research questions are unclear and motivated this research: (a) What is the status of road traffic mortality in China currently and over recent years? and (b) Has road traffic safety in China improved to the extent the police data reflect?

The modified Smeed equation is used to fit the relationship between road traffic mortality and the level of motorization (per capita motor vehicles) for a country or region, helping to portray trends in road traffic mortality and predict mortality from road traffic crashes as that country or region motorizes [12, 13]. To address our research questions mentioned above, we fitted the modified Smeed equation using both police data and health department data from China. We also compared Chinese trends to those of 13 other countries (Belgium, Finland, Greece, Hungary, Ireland, Japan, Lithuania, New Zealand, Portugual, Slovenia, Spain, Poland, and United States). We hypothesized that (1) road traffic mortality in China has not yet peaked at its highest stage; and (2) road traffic mortality in China is not decreasing in the manner reflected by police data.

## Materials and Methods

### Ethical issue

This study uses open access data and does not involve the information of individuals. This study was approved by the Medical Ethics Committee of Central South University.

### Data sources

Road traffic mortality data were obtained from the Ministry of Public Security (police data) and from the National Health and Famly Planning Commission (health data) for China [14, 15]. For police data, we retrieved data collected over a span of 44 years, from 1970 to 2013; data from each year included the number of road traffic deaths, the number of registered motor vehicles, and the size of the population. For health data, we collected road traffic mortality data from 2002 to 2013; health data prior to 2002 are not available to researchers or the public [16].

Police data are reported in the *Road Traffic Accident Statistics Annual Report for China* [17]. The road traffic death data are recorded by traffic police. Local traffic management bureaus within Departments of Public Security regularly summarize the traffic police reports, capturing information about deaths, non-fatal injuries and economic loss from all road traffic crashes. These data are reported through several levels of administration (local, municipality, provincial) and ultimately are compiled by the Ministry of Public Security of China. Police data include deaths that occur within 7 days after the crash [18], a standard that is different from the common operational definition of 30 days in most countries [19, 20]. Replicating the strategy of the World Health Organization [21], we used the recommended standard from the European Conference of Ministers of Transport to translate the fatality data from the 7-day definition to the more standard 30-day definition by multiplying a ratio of 1.08.

Health data were sourced from the *Chinese Health Statistics Yearbook*, which has been released annually since 2002 by the National Health and Family Planning Commission (former name: the Ministry of Health) [16]. Health data are collected through the Chinese Death Cause Registration system, which is estimated based on a sample of about 10% of the total population. Health mortality data are derived from physician-completed death certificates. Families are required to present death certificates to get permission for cremation or burial when a person dies. The death certificates are first submitted to police departments by family members and then forwarded to the municipal, provincial, and national departments of health [15]. Since 2002, health data are coded using standards from the 10^th^ version of International Classification of Diseases (ICD-10). Traffic crash fatality data use the standard of deaths within 30 days following the crash [16].

We also collected data from 13 other countries that offered complete data related to our research purposes from the online library of the Organization for Economic Cooperation and Development (OECD) [22]. The thirteen countries were selected primarily because they had complete data available to match our research purposes; they also cover a range of different types of countries from different regions of the world. Occasional data were missing for some countries (e.g., Lithuania’s traffic crash data before 1989). All 13 countries adopt the 30-days operational definition of road traffic deaths.

### Modified Smeed equation

The original Smeed formula was proposed in 1945 to characterize relations between road traffic mortality and the level of motorization in different countries or regions [13, 23, 24], but it failed to reflect the potential for changing characteristics of road traffic mortality (e.g., an upward stage and peak followed by a downward stage) [25, 26, 27]. To overcome this limitation, several researchers have attempted to improve the Smeed formula [28, 29, 30]. In 2010, Koren et al. proposed a modified equation based on the original Smeed formula as follows [12]:

$$\frac{D}{P}=a\cdot \frac{N}{P}\cdot e^{-b\frac{N}{P}}$$ (1)

In which, *a* and *b* are the parameters to be estimated and *e* refers to the natural constant (approximately 2.7183). When the number of road traffic deaths (*D*), the number of registered motor vehicles (*N*) and the population size (*P*) are given, one can estimate the values of *a* and *b*.

Under a low motorization level, the ratio of *N/P* is close to zero, so the exp(−*b*⋅*N*/*P*) approaches 1 and the population-based fatality rate *D/P* depends largely on the value of *N/P*. In this case, the road traffic fatality rate would increase as the level of motorization rises without considering other factors.

In situations with high motorization level (in other words, when *N/P* is large), exp(−*b*⋅*N*/*P*) begins to play a more important role in the fatality rate *D/P*. In that case, the road traffic fatality rate would not increase as the level of motorization rises; instead, road traffic fatality rates may begin to drop as the result of prevention efforts [31, 32]. Thus, the modified Smeed equation roughly characterizes the development of road traffic mortality relative to the level of motorization.

Eq (1) can be expressed as a generalized linear model (see Eq (2)) and fitted through standard statistical software.

$$Y=\beta_{0}+\beta_{1}\cdot X+\varepsilon$$ (2)

In which

$$\begin{array}{l} Y=\ln\left(\frac{D}{P}\right)-\ln\left(\frac{N}{P}\right)\\ X=\frac{N}{P}\\ \beta_{0}=\ln\left(a\right)\\ \beta_{1}=-b \end{array}$$ (3)

Based on the equation and the values of undetermined coefficients *β*_0_, *β*_1_, *a* and *b*, a curve chart was produced through Matlab2013 to display the change trend of traffic accident fatality rate.

We differentiated Eq (1) and yielded:

$${\left(\frac{D}{P}\right)}^{\prime }={\left(a\cdot \frac{N}{P}\cdot e^{-b\frac{N}{P}}\right)}^{\prime }=a\cdot e^{-b\frac{N}{P}}\cdot \left(1-b\frac{N}{P}\right)$$ (4)

In Eq (4), the derivative varies greatly as $\frac{N}{P}$ changes. When $0<\frac{N}{P}<1/b$, the derivative is higher than zero; when $\frac{N}{P}=1/b$, the derivative equals zero; and when $\frac{N}{P}>1/b$, the derivative is lower than zero. Thus, stationary point $\frac{1}{b}$ is the extreme point of Eq (1). We plugged the extreme point of $\frac{1}{b}$ into Eq (1) to obtain the local maximum value (and also the global maximum value) of road traffic mortality. Then, we named the global maximum value as the predicted maximum mortality (PMM). At the same time, we used the equation to calculate per capita motor vehicles corresponding to PMM.

$$\text{PMM}=\frac{a}{eb}$$ (5)

$$\text{Per capita motor vehicles corresponding to PMM}=\frac{1}{b}$$ (6)

Per capita motor vehicles corresponding to PMM can be used to approximate the performance of road safety management of a country or a region. Generally, low per capita motor vehicles corresponding to PMM indicate effective and efficient responses to the grim situation of road traffic safety [33, 34].

### Statistical analysis

Using Matlab2013, we fitted the modified Smeed equation and tested the statistical significance of models for all countries based on the data we collected. When the absolute value of Studentized Residual was greater than 3.0 or Cook’s Distance was greater than 1.0, the corresponding observed value was regarded as an outlier [35] and excluded from the model fit. After removing these outliers (road traffic mortality of 2011 for Ireland, 2000 for Belgium, 2006 for Slovenia and 1970 for Greece), we fit the modified Smeed equations. *F* test and determination coefficient were used to test the statistical significance of models and assess the goodness-of-fit of models.

## Results

### Model goodness-of-fit

*F* tests show high statistical significance (*p*<0.001) for all fitted models. The coefficients of determination for the fitted models were all greater than 0.83 (ranging from 0.83 to 0.98) (**Table 1**), indicating that the modified Smeed equation properly fit the data of all countries.

**Table 1:** Model fit of modified Smeed equation using the data of China and 13 selected countries.

Country	Data source	Time period	*β*_0_	*β*_1_	*a*	*b*	*R*^2^	*F*	*p*
China^1^	Health data^1^	2002–2013	5.611	-5.742	273.42	5.74	0.852	51.788	<0.001
China^2^	Police data^2^	1970–2013	6.243	-16.392	514.58	16.39	0.831	209.439	<0.001
Belgium	OECD online library^3^	1970–2013	6.241	-5.655	513.34	5.66	0.982	2208.767	<0.001
Finland	OECD online library^3^	1970–2012	6.228	-7.851	506.67	7.85	0.900	197.787	<0.001
Greece	OECD online library^3^	1970–2013	5.131	-3.203	169.17	3.20	0.956	844.840	<0.001
Hungary	OECD online library^3^	1970–2013	5.745	-6.495	312.75	6.50	0.903	379.690	<0.001
Ireland	OECD online library^3^	1970–2013	5.493	-5.315	242.89	5.32	0.899	357.592	<0.001
Japan	OECD online library^3^	1970–2013	4.966	-3.787	143.51	3.79	0.877	299.996	<0.001
Lithuania	OECD online library^3^	1989–2013	5.364	-3.403	213.58	3.40	0.920	265.283	<0.001
New Zealand	OECD online library^3^	1970–2013	6.052	-4.444	424.95	4.44	0.836	203.217	<0.001
Poland	OECD online library^3^	1970,1975,1980,1985,1990–2013	5.221	-3.844	185.12	3.84	0.970	811.433	<0.001
Portugal	OECD online library^3^	1970–2012	6.002	-5.418	404.42	5.42	0.963	1056.563	<0.001
Slovenia	OECD online library^3^	1990–2013	6.478	-6.185	650.75	6.19	0.951	408.871	<0.001
Spain	OECD online library^3^	1970,1980,1990–2013	5.288	-3.998	197.96	4.00	0.859	140.650	<0.001
United States	OECD online library^3^	1970–2013	5.244	-2.709	189.37	2.71	0.847	199.475	<0.001

^1^: Data from the Chinese Health Statistics Yearbook by the National Health and Family Planning Commission of China (former name: the Ministry of Health of China).

^2^: Data from the Road Traffic Accident Statistics Report by the Traffic Management Bureau of the Ministry of Public Security of China.

^3^: Online Library of the Organization for Economic Cooperation and Development (available from: http://internationaltransportforum.org/).

### Road traffic mortality status

We compared the model based on health department data to that based on police data and found differences between them.

**Fig 1:**
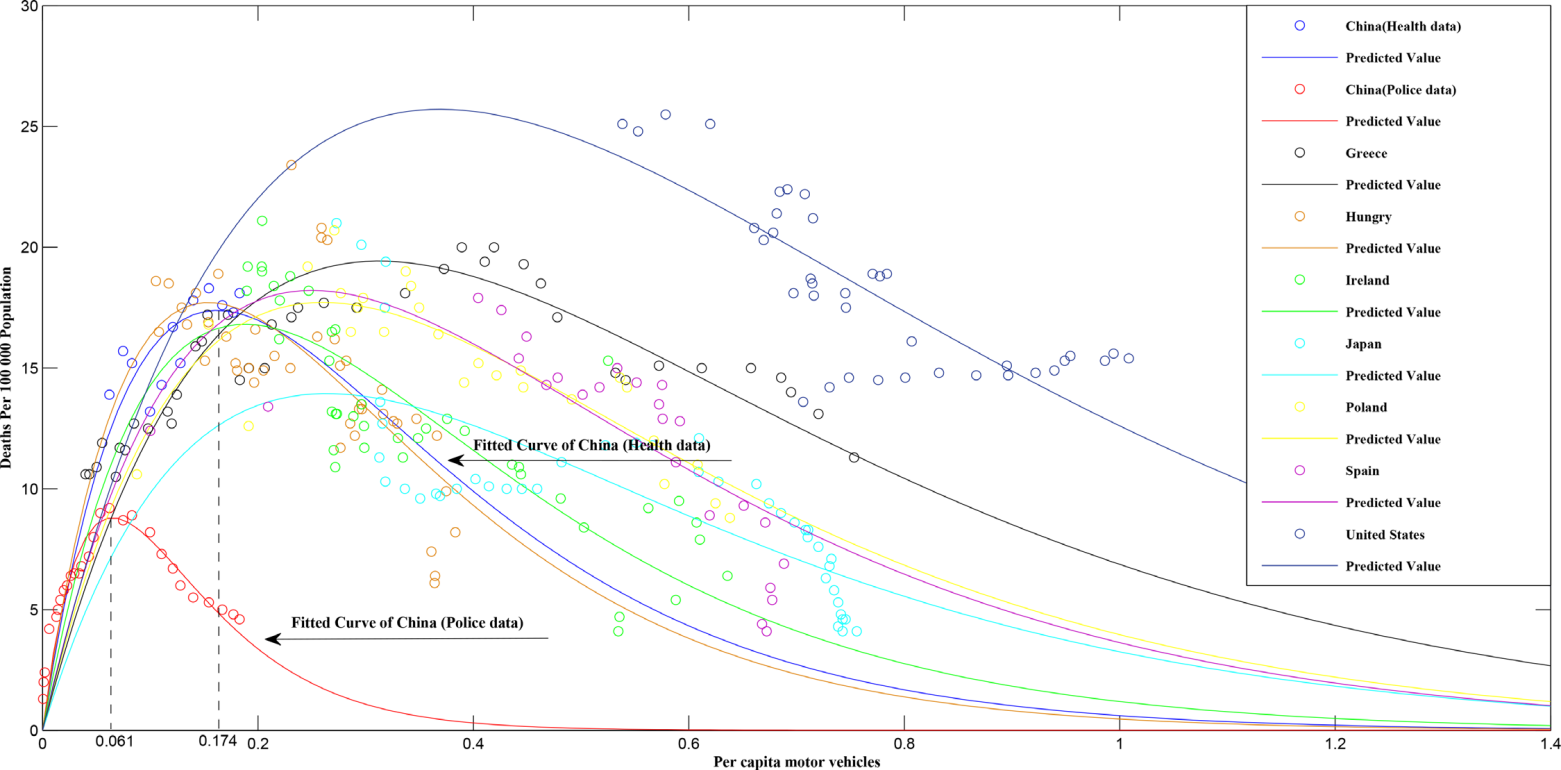
Fitted curves between road traffic mortality and per capita motor vehicles based on modified Smeed equation.

**Fig 2:**
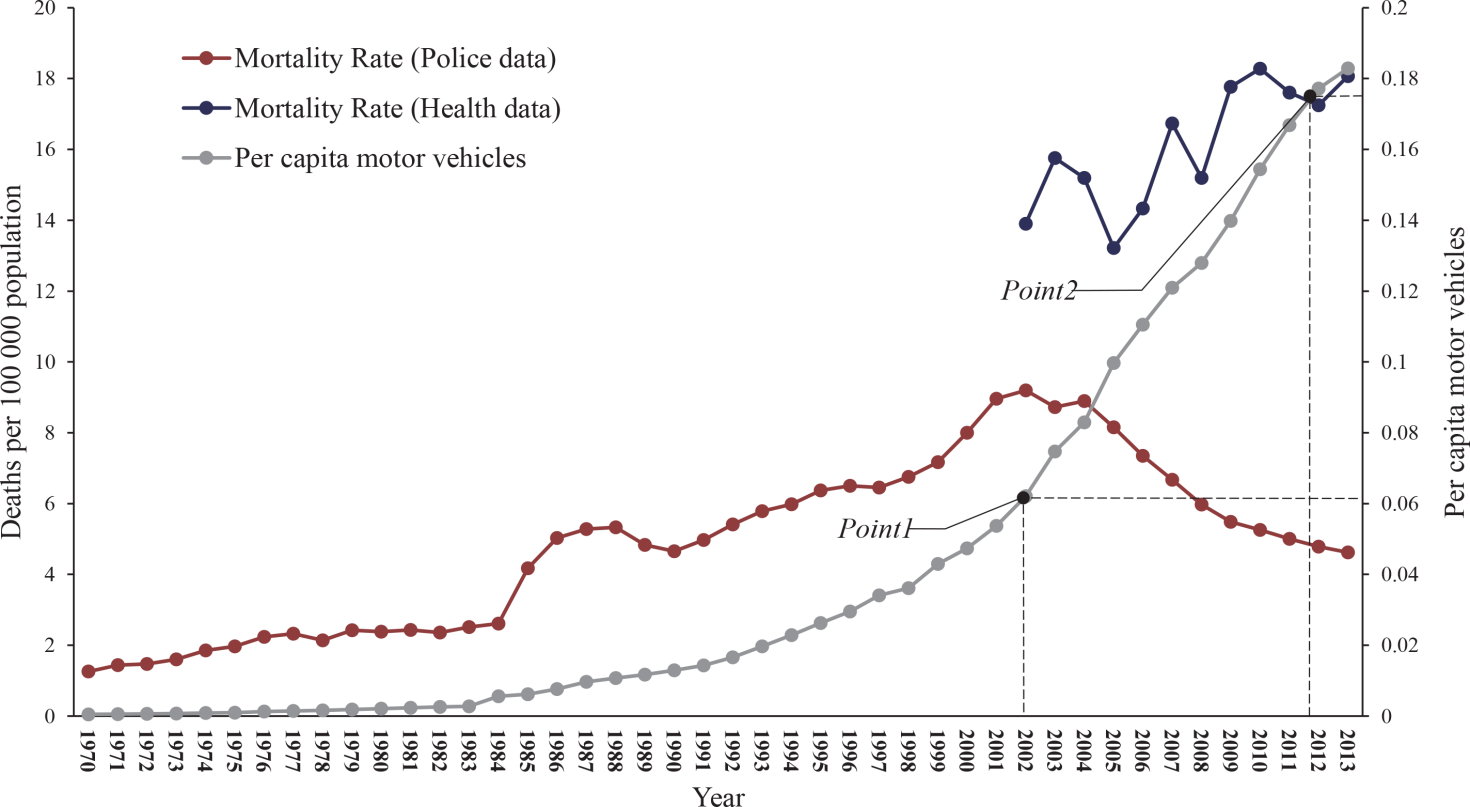
Road traffic mortality from police data and health data and per capita motor vehicles, China, 1970–2013.

According to police data, road traffic mortality peaked at a very low level of motorization (0.06 motor vehicles per person) in 2002. Since 2002, road traffic mortality dropped gradually but substantially, indicating China has successfully managed road traffic crashes and safety is improving substantially (**Fig 1**). On the contrary, health data show that road traffic mortality did not reach a peak until 2012, and at a much higher level of motorization (0.174 motor vehicles per person). These results suggest road traffic mortality in China remains at a high level and safety is poor (**Table 2, Figs 1 and 2**).

**Table 2:** Key results based on the simulation of modified Smeed equation using data from China and 13 selected countries.

Country	PMM (deaths/100,000 persons)	Per capita motor vehicles corresponding to PMM	Year corresponding to PMM
China–health data^1^	17.5	0.174	2012
China–police data^2^	11.5	0.061	2002
Belgium	33.4	0.177	Before 1970
Finland	23.7	0.127	Before 1970
Greece	19.4	0.312	1993–1994
Hungary	17.7	0.154	1977
Ireland	16.8	0.188	1970
Japan	13.9	0.264	Before 1970
Lithuania	23.1	0.294	1996
New Zealand	35.2	0.225	Before 1970
Poland	17.7	0.260	1990–1991
Portugal	27.5	0.185	1979
Slovenia	38.7	0.162	Before 1970
Spain	18.2	0.250	1980–1990
United States	25.7	0.369	Before 1970

PMM: Predicted maximum mortality based on the modified Smeed equation.

^1^: Data from the Chinese Health Statistics Yearbook by the National Health and Family Planning Commission of China (former name: the Ministry of Health of China).

^2^: Data from the Road Traffic Accident Statistics Report by the Traffic Management Bureau of the Ministry of Public Security of China.

### China’s performance in road safety compared to 13 other countries

The PMM in China was 11.5 per 100,000 population according to the police data and 17.5 per 100,000 population according to the health data. The PMM of the other 13 countries ranged from 13.9 per 100,000 population in Japan to 38.7 per 100,000 population in Slovenia. The level of motorization at which the 13 countries reached PMM varied from 0.127 motor vehicles per person in Finland to 0.369 motor vehicles per person in the United States (**Table 2**), figures that can be compared to the results of 0.061 from police data in China and 0.174 from health data in China. Given these data, it seems likely the health data from China are a better approximation of real-life crashes than the police data, as the health data indicate both a PMM and a peak in crashes per capita motor vehicles that fall in the middle of the range of the other nations whereas the police figures are well below the minimum values among the other 13 nations.

## Discussion

### Validity of Modified Smeed equation

Statistical results indicate the models we fit based on the modified Smeed equation characteractize well the association of road traffic mortality with the level of motorization in China and 13 other countries. The results generally agree with previous reports [12, 13] and validate use of the simulation results to examine road traffic mortality trends in China.

### Policy implications

Our findings present comparative evidence on the two key research questions that we proposed, what is the status of road traffic mortality in China and has road traffic safety in China improved to the extent the police data reflect? Although models based on both police data and health department data from China fit the reported data, the model based on health data provides results that are much more consistent with data from other countries.

China’s first significant legislation on road safety was released in 2004 [36]; that legislation dictated required actions to reduce road traffic crashes in multiple governmental departments and outlined a series of required criteria related to the design of vehicles, roads, and road traffic control. Despite the passing of that legislation, however, road traffic safety management in China remains inferior to most developed countries [18, 37, 38, 39, 40, 41], and in need of continued attention. The rapidly improving patterns of road traffic mortality reflected by the police data, which imply much better performance in road traffic safety management, appear unlikely to be valid.

Instead, according to the simulation results from the health data, road traffic mortality in China remains at a very high level currently and has not yet entered a steady decreasing period. These results should be disseminated to Chinese policy-makers and the public, as they contrast substantially with results from police data that currently have a greater influence on decision-making related to road traffic safety than health data.

Equally important, China should systematically review existing data sources and develop a method to make full use of all available data to obtain reliable and valid road traffic safety statistics. Even the health data have limitations that could be overcome (e.g., failure to include details of road traffic crashes; concern about underreporting and misclassification) [14, 42, 43]. The serious underestimation of road traffic mortality rates by police data may have contributed to the failure to pass additional legislation or amend current legislation, and policy-makers should heed scholarly findings to use accurate data to understand the scope of the traffic safety challenges and support policy decision-making in China.

### Limitations of this study

One limitation of this study is the quality of data used. A variety of factors may influence the quality of the data we used, including incomplete reporting and misclassification of road traffic crashes and other diseases and injuries. Another limitation is the mathematical properties of the modified Smeed equation, which depends on the co-occurrence of an increasing and then decreasing stage of road traffic mortality. The equation fits any road traffic mortality series showing an increasing stage and then a decreasing stage but does not have the capacity to fit other trends.

## Conclusions

Our simulation results suggest the health data from China more accurately portray real traffic mortality data than the police data, and China remains at a stage of very high road traffic mortality. National and local efforts should be enhanced to address the challenge of traffic safety in China. Part of these efforts should be efforts to obtain and use accurate data about road traffic injuries and mortalities. The police data that are currently used may not offer such validity.
